# Primary Renal Neuroendocrine Tumour Presenting as an Enormous Cystic Lesion

**DOI:** 10.5146/tjpath.2017.01418

**Published:** 2020-01-15

**Authors:** Azfar Neyaz, Zafar Neyaz, Nuzhat Husain, Vani Gupta

**Affiliations:** Dr. Ram Manohar Lohia Institute of Medical Sciences, Department of Pathology, Lucknow, India; Sanjay Gandhi Postgraduate Institute of Medical Sciences, Department of Radiodiagnosis, Lucknow, India

**Dear Editor,**

**Figure 1:**
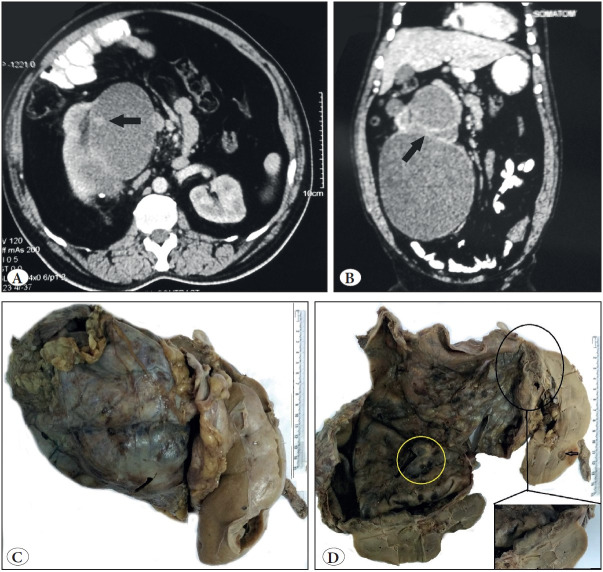
CECT: **A)** Axial: cystic mass arising from upper pole with solid enhancing nodular projection (arrow). **B)** Coronal reconstruction: thick internal septae (arrow). **C)** Total nephrectomy specimen showing large cystic tumor. **D)** Well-defined renal interface (black circle), extrarenal extension with cortical cyst (arrow) and thickened mural nodule (yellow circle)

**Figure 2:**
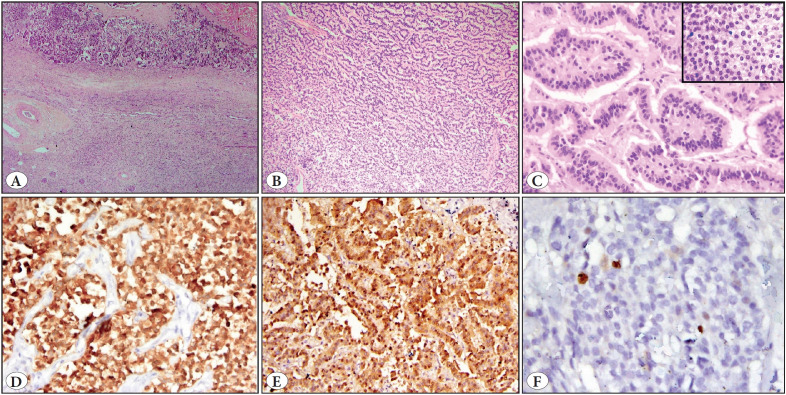
**A)** Tumor with adjoining unremarkable renal parenchyma (H&E; x40). **B)** Tumor cells arranged in cords, trabeculae and ribbons (H&E; x100). **C)** Monomorphic tumor cells with round uniform nuclei with speckled chromatin (inset) (H&E; x200). **D)** Positive synaptophysin expression (IHC; x200). **E)** Positive chromogranin-A expression (IHC; x200). **F)** Ki-67 proliferation index <2% (IHC; x400).

Primary Renal Neuroendocrine tumors (NETs) are extremely rare in the kidney, with fewer than 100 cases reported (1). NETs usually present as solid tumors with or without cystic components. We report a rare presentation of a well-differentiated neuroendocrine tumor as a large completely cystic lesion in the right kidney without any evidence of metastasis or carcinoid syndrome. Very few case reports of NETs of such a large size have, so far, been reported in literature. A 47-year-old man presented with an incidentally detected large non-tender and mobile mass in the right iliac fossa. USG showed a large 23.5 x 14.0 cm cystic space-occupying lesion with mixed echoes abutting the right kidney. Contrast enhanced computerized tomography (CECT) showed a predominantly cystic mass arising from the upper pole of the right kidney with a small solid enhancing nodular projection on the inner wall. Thick internal enhancing septations and multiple calcific foci were also present in the wall. The lesion was seen to compress the right pelvicalyceal system with moderate hydronephrosis (Figure 1). Laboratory investigations were within normal limits. The Renal scan showed mildly impaired right kidney function. The patient underwent a radical right nephrectomy. On gross evaluation, a large circumscribed cystic tumor arising from the upper pole of the kidney partly within the renal parenchyma with extension outside the kidney capsule was identified. The lesion was completely cystic with a thick nodular hemorrhagic wall (Figure 1). The renal interface was well defined and separated by a fibrous capsule (Figure 1 inset). Histology had a characteristic trabecular and ribbon–like pattern. The neoplastic cells had rounded regular nuclei with stippled chromatin and minimal mitotic activity (<2/10 HPF) was observed (Figure 2). Tumor cells expressed pancytokeratin, chromogranin A and synaptophysin on immunohistochemistry (Figure 2) and had a low proliferation index with Ki-67 of less than 2% (Figure 2). A diagnosis of well-differentiated neuroendocrine tumor (Grade 1) was rendered. Subsequent whole body scan for metastatic or primary lesions did not reveal any active foci. The patient has been well during a 12 months follow up.

Well-differentiated neuroendocrine tumor in the kidney is extremely rare (1). Renal NETs are usually solid and may show a cystic component. Our case was completely cystic with a size of 23.5 cm in diameter along with a mural nodule, which is a rare presentation of renal neuroendocrine tumors. The pathogenesis of renal neuroendocrine tumors is controversial. They have been reported to arise in congenital anomalies like polycystic kidney disease, horseshoe kidney and mature teratoma (2). Well-differentiated neuroendocrine tumors are incidentally detected in 25–30% of the cases. The NET in our case was also incidentally discovered during an ultrasound of abdomen despite its large size (2). Calcification in the renal cyst wall may occur. Metastasis is a rare event and may occur to the regional lymph nodes, liver, bone, breast, and lung (3). Metastatic potential is higher in solid tumors, larger than 4 cm, outside the renal capsule and with a mitotic rate of more than 1/10 high power fields (1). Positron emission tomography (PET-CT) and octreotide scintigraphy are useful for diagnosis, staging, and monitoring after treatment for the development of recurrence or metastasis of neuroendocrine tumors (4).

Well-differentiated neuroendocrine tumors should be considered in the differential diagnosis of predominantly cystic lesions in the kidney. Other lesions presenting as renal cysts include clear cell renal cell carcinoma (RCC), especially in cases with low nuclear grades, papillary RCC, chromophobe RCC, collecting duct carcinoma, or even oncocytoma and they may all occasionally demonstrate focal to extensive cystic formation. Simple benign cysts are commonest and may become complicated in case of hemorrhage, infection and ischemia. On radioimaging, cystic NET tends to be hypodense, does not usually enhance in arterial phases and may show calcification. Large size and extrarenal extension herald metastatic potential despite low-grade morphology and such patients should undergo rigorous oncologic surveillance after nephrectomy.
